# Alcohol consumption and associated factors among middle-aged and older adults: results from China Health and Retirement Longitudinal Study

**DOI:** 10.1186/s12889-022-12718-8

**Published:** 2022-02-15

**Authors:** Qianqian Wang, Yanzhuo Zhang, Chengai Wu

**Affiliations:** 1 Department of Epidemiology and Biostatistics, Beijing Research Institute of Traumatology and Orthopaedics, Beijing Jishuitan Hospital, 100035 Beijing, China; 2 Department of Molecular Orthopaedics, Beijing Research Institute of Traumatology and Orthopaedics, Beijing Jishuitan Hospital, 100035 Beijing, China

**Keywords:** Alcohol drinking, China, Cross-sectional studies

## Abstract

**Background:**

This study aimed to investigate alcohol consumption and associated factors in middle-aged and older adults.

**Materials and methods:**

We included 15,942 participants (7,384 men, 8558 women) with age range of 45-101 years from the 2011 baseline survey of the China Health and Retirement Longitudinal Study. Variables related to alcohol status and other potential risk factors were selected for analysis. Logistic regression analysis was used to analyze the factors associated with drinking.

**Results:**

There was a significantly higher proportion of current drinkers among men than women (36.42% ± 0.86% vs. 3.73% ± 0.27%). Among the current drinkers, proportions of binge drinking, heavy drinking and daily drinking were 38.2, 53.0, 57.5% for men and 10.9, 37.2, 36.2% for women, respectively. Factors significantly associated with current drinking were age, health situation, smoking, geographic region, work status and social activities among men, and age, smoking, geographic region and work status among women. The favorite type of alcohol was spirits for both men and women. The type of beverage intake was also related socio- cultural-demographic factors as mentioned above.

**Conclusions:**

Alcohol consumption behaviors and type of beverage was significantly influenced by socio- cultural-demographic factors. The socio-cultural-demographic factors affecting alcohol patterns should be further focused on to promote the development of alcohol control strategies.

## Introduction

Alcohol use disorders have been reported to be the most prevalent of all substance use disorders [1]. It was estimated that 992 million disability-adjusted life years (DALYs) and 4.2% of all DALYs were attributable to alcohol use in 2016 [1]. The burden attributable to alcohol was primarily from cirrhosis, transport injuries and cancer. It has also been suggested that the alcohol-attributable burden is highest in countries with either a low or middle–high socio-demographic index [1].

Alcohol drinking traditionally plays an important role in social activities in China. Drinking is considered to improve the atmosphere at festivals and ceremonies, and social drinking is encouraged as a way to build good relations in business [2]. Steady increases have been observed in alcohol production, availability and per capita consumption in China over recent decades because of rapid economic development and urbanization. These have led to increased levels of alcohol-related harm [3]. China faces an urgent need to implement a more rigorous alcohol policy to control the prevalence of alcohol-related problems [4].

Appropriate public health policies require understanding of which population groups are most affected by harmful alcohol use. In the last decades, several cross-sectional studies have explored the patterns of alcohol consumption in China. For example, Li et al. used data from the China Chronic Disease and Risk Factor Surveillance in 2007 to describe drinking patterns among Chinese residents aged 15–69 years [5]. However, they had no data on older people and insufficient information about alcohol-related risk factors. Lee et al. described alcohol consumption behaviors and alcohol dependence among older adults (aged 60 years and over) in China using the Chinese Longitudinal Healthy Longevity Survey (CLHLS) waves from 2000 to 2014 [6]. However, this study failed to explore the alcohol type and amount of alcohol consumed, and only reported the drinking status of older people. Using the 2010–2012 China National Nutrition and Health Survey, Li et al. explored drinking status and associated factors in adults aged 18 years and over and reported the rates of drinking, excessive drinking and harmful drinking [7]. However, it failed to explore drinking patterns. Considering the multi-dimensional characteristics of alcohol consumption measurement, researchers need to inspect covariation of quantity, frequency alcohol type and drinking pattern that contributes to causal inference between alcohol consumption and medical outcomes, and developing drinking guidelines.

To fill those gaps, we analyzed the data from the 2011 China Health and Retirement Longitudinal Study (CHARLS) baseline survey that included subjects aged ≥ 45 years and further simultaneously considered variables related to alcohol type, drinking frequency and volume, and drinking pattern to explore alcohol consumption status and examine the associations of potential risk factors with these behaviors.

## Methods

### Study sample and procedures

The CHARLS is an ongoing nationally representative longitudinal cohort study in China conducted by the National School of Development at Peking University. Residents aged 45 years and older and their spouses were interviewed at their homes, and data on socioeconomic and health status were collected using standardized questionnaires. A detailed description of the CHARLS has been published elsewhere [8]. Briefly, samples were selected using four stage stratified probability sampling. In the first stage, 150 counties within 28 provinces were randomly selected with a systematic sampling. In the second stage, probability-proportional-to-size sampling technique was used to choose 3 rural villages or urban communities (primary sampling units, PSUs) in each county. In the third stage, 80 households which contained residents aged ≥45 years were selected from each PSU using simple random sampling. In the final stage, one age-eligible person from the selected household were asked to participate in the survey. The baseline survey was conducted between June 2010 and March 2012. The respondents are followed every 2 years, and information collected through face-to-face computer-assisted personal interviews (CAPIs) with interviewers trained at Peking University by CHARLS staff members. Physical examinations were also carried out at the interviewees’ homes by trained interviewers. Information collected included demographic factors, socioeconomic status, and medical history.

In this study, we used the baseline survey, conducted between June 2011 and March 2012, which involved 17,708 respondents. We selected those aged 45 years and older. We removed anyone who had not provided full responses to the questions of interests, leaving data from 15,942 individuals (male: 7384; female: 8558) for analysis.

### Measures

#### Alcohol consumption

In the baseline survey questionnaire, participants were asked how often they had drunk alcohol during the past year (never; less than once a month; ≥ once a month). Those who drank ≥ once a month in the last year were defined as ‘current drinkers’. Non-current drinkers were further asked if they had ever drunk alcoholic beverages in the past and how often (never; less than once a month; ≥ once a month). Those who never drank and had drank less than once a month in the past were considered as ‘abstainers’; those who previously drank ≥ once a month were defined as ‘former drinkers’; those who drank less than once a month in the last year and in the past were defined as ‘occasional drinkers’.

Current drinkers were further asked what type of alcoholic beverages (spirits, beer, or wine) and what amount they had most recently drunk (by 220 ml mugs or 550 ml bottles for beer, and number of 50 ml “liang” for wines and spirits) at the last drinking, and how often in the last 12 months (once a month, 2–3 times a month, once a week, 2–3 times a week, 4–6 times a week, once a day, twice a day, more than twice a day). They also indicated the age when start drinking and whether they drank first thing in the morning to steady their nerves or get rid of a hangover. We converted measures of frequency and number of alcoholic drinks consumed into units of pure alcohol consumed per week. The following alcohol content by volume (v/v) was used for this calculation: beer 3.9%, wine 12%, spirits 53% [9]. One drink (unit) was defined as 14 g of pure alcohol consumed [10]. Current drinkers were classified into 0–13.9 units per week, 14.0–27.9 units per week and ≥ 28 units per week for men and 0–6.9 units per week and ≥ 7.0 units per week for women. Heavy drinkers were those who drank more than 14.0 drinks per week for men and more than 7.0 drinks per week for women [10]. A “binge” was defined as consuming 5 or more drinks (male), or 4 or more drinks (female), in about 2 h [11].

#### Covariates

We included age in years, living in urban or rural areas, education, rural-urban status, smoking status, depression, working status, social activities and sleep time as potential control variables in logistic regression analyses. We categorized participants into seven age groups (45–49 years, 50–54 years, 55–59 years, 60–64 years, 65–69 years, 70–74 years, and ≥ 75 years) and categorized their area of residence into seven regions, East (one city [Shanghai] and six provinces [Shandong, Jiangsu, Zhejiang, Fujian, Anhui, and Jiangxi]), North (two cities [Beijing and Tianjin] and three provinces [Hebei, Shanxi, and Inner Mongolia]), North-East (three provinces [Liaoning, Jilin, and Heilongjiang]), North-West (four provinces [Shaanxi, Gansu, Qinghai, and Xinjiang]), Central (three provinces [Henan, Hubei, and Hunan]), South-Central (two provinces [Guangdong, and Guangxi]), and South-West (one city [Chongqing] and three provinces [Sichuan, Guizhou, and Yunnan]) [12]. Individuals living in Hainan, Ningxia, Taiwan, and Tibet were not included in this survey. All participants were classified as either urban or rural residents. Education level was divided into four categories: no formal education, elementary school, middle/high school, and college degree or higher. Self-assessed health was divided into five categories: excellent, very good, good, fair and poor. Being a smoker was defined as smoking more than 100 cigarettes over the course of a lifetime [13]. The measure of depressive symptoms was based on responses to the 10-item version of the Center for Epidemiologic Studies Short Depression Scale (CES - D). Each item had four response options coded from 0 to 3. The total score was the sum for all 10 items. A dichotomous variable was created using a score of 10 or more to indicate the presence of depressive symptoms [14]. Current working was defined as “engaging in agricultural work for more than 10 days in the past year” or “working for at least one hour last week”. Participating in social activities was defined as “Have done any of these activities in the last month”. Total sleep time per day was calculated by adding the sleep time at night to the duration of a nap after lunch. Sleep time was divided into three categories: <6.0 h, 6.0~7.9 h and ≥8.0 h. Details of the items was listed by BASELINE QUESTIONNAIRE for CHARLS [13].

### Statistical analysis

Continuous variables were shown as mean and standard deviation (SD), and categorical variables as percentage and corresponding standard error. All statistical analyses used SAS version 9.2, with adjustments for sample weights and survey design. Proc Surveyfreq procedure was used to calculate sex-specific prevalence of current drinking for each risk factor. Proc Surveylogistic procedure was used to examine the association between each risk factor and the prevalence of current and binge drinking. Both procedures considered the complex survey design and the nonresponse rate for the CHARLS survey when estimating the prevalence, prevalence odds ratio (OR), and corresponding standard errors and 95% confidence intervals (CIs), respectively. The sex-specific associations between the potential risk factors and alcohol consumption were tested by the statistical models adjusting for demographic characteristics (age groups, rural-urban status, marital status and region), and living status, smoking status, self- comment of health, depression, education, working status, participating in social activities and sleep time). All statistical tests were 2-sided and a *P* value less than 0.05 was considered statistically significant.

## Results

### Participants and current alcohol consumption

A total of 15,942 people (7,384 men and 8,558 women) with age range of 45-101 years were included in the analysis. Their mean age was 59.77 ± 9.68 years for men and 59.17 ± 10.06 years for women.

Table 1 shows the estimated prevalence rates of current drinking. The proportion of current drinkers was significantly higher among men than women (36.42% ± 0.86% vs. 3.73% ± 0.27%). In men, the prevalence of current drinking was higher at younger ages, in married subjects, in north-east areas, and in those with higher levels of education. In women, the prevalence was higher in rural areas and those living in the south-west region.

**Table 1:** The prevalence of current drinking stratified by different characteristics

		Men					Women			
	N	Prevalence	SE	χ^2^	*P* value	N	Prevalence	SE	χ^2^	*P* value
**Age (years)**										
45~49	1355	39.64	1.98	84.128	<0.001	1836	2.98	0.43	7.351	0.290
50~54	1099	41.32	1.89			1290	3.80	0.63		
55~59	1503	42.73	2.09			1746	4.23	0.51		
60~64	1294	38.07	1.59			1402	4.64	0.62		
65~69	842	32.58	2.26			864	3.79	0.66		
70~74	647	24.09	2.15			637	3.48	0.73		
≥75	644	22.62	2.11			783	3.16	0.71		
**Rural-urban status**										
Rural	5613	36.72	1.07	0.257	0.613	6740	4.10	0.33	6.332	0.012
Urban	1721	35.55	1.89			1760	2.61	0.43		
**Marital status**										
Separated/divorced/widowed/never been married	705	33.53	3.33	0.808	0.369	1403	3.74	0.60	0.000	0.995
Married	6678	36.74	0.90			7155	3.73	0.29		
**Education**										
Illiteracy	971	30.44	1.95	5.968	0.113	3512	4.05	0.41	6.992	0.072
Primary school	3240	36.64	1.17			2948	3.84	0.40		
Junior high school	1944	37.48	1.92			1311	2.41	0.41		
Senior high school or Graduate	1222	38.09	2.60			776	4.25	0.85		
**Geographic region**										
Central	1132	35.85	2.25	74.582	<0.001	1311	3.39	0.70	44.792	<0.001
East	2243	43.86	1.35			2593	4.84	0.53		
North-central	1065	34.69	2.29			1190	3.13	0.69		
North-east	549	48.74	2.07			653	3.54	0.80		
North-west	561	20.78	2.08			608	1.13	0.43		
South	566	19.82	4.81			780	0.91	0.32		
South-west	1268	38.71	1.98			1423	6.05	0.96		
**Total**	7,384	36.42	0.86	-	-	8,558	3.73	0.27	-	-

*SE *standard error

Among the current drinkers, proportions of binge drinking, heavy drinking and daily drinking were 38.2, 53.0, 57.5% for men and 10.9, 37.2, 36.2% for women, respectively. Men started drinking at significantly younger ages than women (23.24 ± 8.22 years vs. 28.25 ± 13.77 years). In total, 4.88% of current drinkers drank first thing in the morning to steady their nerves or get rid of a hangover. Both men and women living in rural areas had a higher proportion of “morning drinking” than those in urban areas (men: 6.11% vs. 1.83%; women: 2.89% vs. 0.00%).

### Drinking behavior

The drinking status by factors is shown in Tables 2 and 3. Among men, there were significant differences in drinking status by age, marital status, education, smoking status, depression, working status and sleep time. There were more occasional drinkers among older people, and those with depression or poor health. Married drinkers seemed to be more likely to quit drinking. There were more former drinkers among participants with high levels of education. There was a higher proportion of heavy drinkers among those living in the north-east. Those who were working were more likely to be drinkers and heavy drinkers.

**Table 2:** Alcohol consumption status (percentage and corresponding standard error) by socio-demographic characteristics and health factors in men

		Alcohol drinking category							
	N	Lifetime abstainer	Former drinkers	Occasional drinkers	0~13.9 dks/wk	14.0~27.9 dks/wk	≥28 dks/wk	χ^2^	*P* value
**Age (years)**									
45~49	1355	36.45 (2.06)	15.88 (2.03)	8.03 (2.22)	20.67 (1.47)	7.63 (1.49)	11.34 (1.05)	140.74	<0.001
50~54	1099	38.33 (2.00)	13.86 (2.11)	6.49 (0.82)	20.22 (1.46)	7.24 (0.80)	13.86 (1.31)		
55~59	1503	37.95 (1.62)	10.77 (1.13)	8.55 (0.88)	21.23 (1.99)	9.27 (1.33)	12.24 (1.07)		
60~64	1294	38. (1.52)	10.11 (1.03)	13.83 (1.15)	16.35 (1.22)	7.85 (0.96)	13.86 (1.21)		
65~69	842	40.21 (2.60)	11.9 (3.70)	15.31 (1.53)	14.97 (1.68)	6.64 (0.93)	10.97 (1.21)		
70~74	647	50.26 (2.46)	7.54 (1.25)	18.11 (1.83)	11.64 (1.79)	5.12 (0.87)	7.33 (1.17)		
≥75	644	56.49 (3.10)	8.53 (2.88)	12.36 (1.45)	12.62 (1.79)	5.19 (0.87)	4.81 (0.92)		
**Rural-urban status**									
Rural	5613	40.83 (1.18)	11.6 (0.79)	10.84 (0.76)	16.92 (0.65)	7.53 (0.45)	12.28 (0.64)	7.42	0.191
Urban	1721	41.29 (2.07)	12.05 (1.36)	11.12 (1.08)	20.09 (1.28)	6.79 (1.77)	8.67 (1.02)		
**Marital status**									
Separated/divorced/widowed/ never been married	705	47.52 (2.34)	7.63 (1.28)	11.31 (1.66)	15.56 (2.06)	9.1 (2.22)	8.87 (1.39)	14.45	0.013
Married	6678	40.11 (1.08)	12.25 (0.85)	10.9 (0.64)	18.05 (0.67)	7.15 (0.46)	11.54 (0.57)		
**Living alone**									
No	6290	40.21 (0.99)	11.55 (0.85)	10.61 (0.50)	18.28 (0.64)	7.42 (0.47)	11.93 (0.59)	6.88	0.230
Yes	1093	44.44 (3.07)	12.72 (2.31)	12.6 (2.36)	15.29 (2.55)	7.09 (1.63)	7.87 (1.07)		
**Education**									
Illiteracy	971	47.72 (2.28)	8.85 (1.12)	12.99 (1.39)	12.89 (1.41)	7.71 (1.03)	9.83 (1.09)	34.35	0.003
Primary or junior high school	3240	41.17 (1.24)	10.07 (1.12)	12.13 (0.69)	16.97 (0.81)	7.29 (0.55)	12.38 (0.77)		
Senior high school	1944	39.28 (1.72)	13.38 (1.30)	9.86 (1.69)	19.53 (1.66)	6.61 (0.59)	11.35 (0.96)		
Graduate	1222	38.38 (2.42)	14.81 (2.01)	8.71 (1.23)	20.2 (1.37)	8.4 (2.51)	9.49 (1.30)		
**Smoking**									
No	2001	56.63 (1.83)	11.65 (1.67)	7.69 (0.74)	14.26 (1.12)	4.18 (0.55)	5.58 (0.58)	147.89	<0.001
Yes	5383	34.77 (1.05)	11.78 (0.74)	12.2 (0.78)	19.2 (0.80)	8.6 (0.79)	13.45 (0.72)		
**Self- Comment of Your Health**									
Excellent	297	30.86 (4.19)	11.05 (2.17)	6.79 (1.76)	24.54 (6.50)	12.11 (5.52)	14.66 (3.18)	90.52	<0.001
Very good	987	39.54 (2.36)	10.87 (1.18)	5.68 (0.86)	18.93 (1.46)	9.75 (2.15)	15.24 (1.31)		
Good	2436	38.81 (1.63)	13.39 (1.43)	9.12 (1.40)	19.41 (1.12)	7.06 (0.60)	12.2 (0.84)		
Fair	2545	42.08 (1.53)	12.45 (1.44)	12.32 (0.83)	16.39 (1.02)	6.8 (0.61)	9.95 (0.73)		
Poor	1117	47.71 (1.94)	7.33 (0.99)	18.53 (1.53)	14.07 (1.24)	5.42 (0.84)	6.95 (0.88)		
**Region**									
Central	1132	40.49 (2.34)	11.5 (1.25)	12.16 (1.19)	19.49 (1.61)	6.94 (0.98)	9.42 (1.07)	162.30	<0.001
East	2243	35.8 (1.49)	9.8 (1.27)	10.54 (0.89)	19.81 (1.21)	8.54 (0.74)	15.51 (1.19)		
North-central	1065	45.75 (1.99)	10.85 (1.49)	8.71 (1.07)	18.69 (1.22)	6.22 (0.88)	9.78 (1.52)		
North-east	549	31.98 (2.48)	8.43 (1.37)	10.86 (1.89)	24.66 (1.66)	6.98 (1.22)	17.1 (2.51)		
North-west	561	51.12 (2.92)	18.32 (1.71)	9.78 (1.70)	16.83 (2.01)	2.17 (0.62)	1.78 (0.57)		
South	566	52.87 (6.95)	19.17 (3.36)	8.13 (3.56)	9.01 (1.59)	7.81 (3.79)	2.99 (0.91)		
South-west	1268	37.19 (2.01)	8.94 (0.99)	15.16 (1.39)	15.76 (1.44)	8.87 (1.08)	14.09 (1.21)		
**Depression**									
No	4578	39.00 (1.11)	11.12 (0.89)	8.66 (0.50)	19.86 (0.86)	8.21 (0.92)	13.14 (0.73)	42.05	<0.001
Yes	2035	42.31 (1.53)	8.37 (0.75)	14.3 (0.98)	17.75 (1.06)	7.05 (0.67)	10.22 (0.81)		
**Working status**									
NO	2021	48.08 (1.36)	9.35 (0.92)	15.15 (1.16)	14.44 (1.00)	6.16 (0.84)	6.83 (0.71)	105.92	<0.001
Yes	5363	37.53 (1.21)	12.87 (0.88)	8.95 (0.73)	19.40 (0.79)	7.93 (0.59)	13.32 (0.68)		
**Social activities**									
No	3445	42.98 (1.43)	10.46 (1.58)	10.53 (0.69)	17.25 (1.07)	6.94 (0.50)	11.84 (0.74)	5.79	0.327
Yes	3939	39.24 (1.18)	12.77 (0.95)	11.27 (0.99)	18.26 (0.78)	7.70 (0.99)	10.76 (0.69)		
**Sleep time**									
<6.0 h	1449	39.32 (1.86)	8.74 (0.87)	13.81 (1.07)	18.75 (1.34)	7.61 (0.82)	11.77 (1.08)	25.13	0.005
6.0~7.9 h	2532	38.21 (1.42)	11.39 (0.85)	9.21 (0.82)	21.33 (0.91)	8.77 (1.49)	11.09 (0.87)		
≥8.0 h	2977	41.36 (1.42)	11.14 (1.37)	9.51 (0.63)	17.47 (1.15)	7.35 (0.54)	13.17 (0.81)		

Drs/wk: drinks/week

**Table 3:** Alcohol consumption status (percentage and corresponding standard error) by socio-demographic characteristics and health factors in women

	Alcohol drinking category							
	N	lifetime abstainer	Former drinkers	Occasional drinkers	0~ 6.9dks/wk	≥7.0 dks/wk	χ^2^	*P* value
**Age (years)**								
45~49	1836	88.48 (1.61)	7.86 (1.58)	0.68 (0.19)	2.18 (0.36)	0.81 (0.20)	80.62	<0.001
50~54	1290	86.54 (1.68)	8.22 (1.65)	1.44 (0.43)	2.64 (0.47)	1.16 (0.42)		
55~59	1746	89.79 (0.93)	3.73 (0.58)	2.25 (0.42)	2.57 (0.40)	1.66 (0.29)		
60~64	1402	88.31 (1.08)	4.25 (0.64)	2.8 (0.50)	2.42 (0.46)	2.21 (0.42)		
65~69	864	88.77 (1.24)	4.36 (0.92)	3.08 (0.57)	2.54 (0.58)	1.25 (0.35)		
70~74	637	90.21 (1.33)	3.76 (0.97)	2.54 (0.64)	2.34 (0.62)	1.14 (0.38)		
≥75	783	91.19 (1.17)	2.99 (0.63)	2.66 (0.69)	1.35 (0.46)	1.81 (0.56)		
**Rural-urban status**								
Rural	6740	88.96 (0.66)	5.07 (0.48)	1.87 (0.21)	2.43 (0.23)	1.68 (0.20)	7.50	0.112
Urban	1760	88.81 (1.41)	6.24 (1.26)	2.34 (0.50)	1.92 (0.36)	0.69 (0.19)		
**Marital status**								
Separated/divorced/widowed/ never been married	1403	89.43 (1.02)	3.9 (0.59)	2.93 (0.64)	1.93 (0.39)	1.81 (0.40)	13.02	0.011
Married	7155	88.75 (0.64)	5.71 (0.55)	1.8 (0.18)	2.4 (0.23)	1.33 (0.16)		
**Living alone**								
No	6617	88.58 (0.68)	5.8 (0.60)	1.85 (0.19)	2.38 (0.24)	1.38 (0.17)	7.88	0.096
Yes	1940	89.74 (0.94)	4.14 (0.52)	2.47 (0.50)	2.11 (0.34)	1.53 (0.33)		
**Education**								
Illiteracy	3512	89.02 (0.87)	4.58 (0.71)	2.35 (0.30)	2.32 (0.30)	1.74 (0.27)	66.84	<0.001
Primary or junior high school	2948	89.4 (0.76)	4.9 (0.50)	1.85 (0.29)	2.38 (0.31)	1.46 (0.24)		
Senior high school	776	92.48 (0.85)	3.87 (0.75)	1.23 (0.37)	1.65 (0.36)	0.77 (0.20)		
Graduate	8547	81.35 (2.41)	11.9 (2.56)	2.5 (0.69)	3.11 (0.68)	1.14 (0.39)		
**Smoking**								
No	7868	89.53 (0.60)	5.43 (0.50)	1.86 (0.21)	2.12 (0.20)	1.06 (0.14)	109.68	<0.001
Yes	689	80.78 (1.71)	4.67 (0.91)	3.99 (0.82)	4.67 (0.90)	5.89 (1.02)		
**Self- Comment of Your Health**								
Excellent	227	87.6 (2.50)	4.98 (1.37)	3.07 (1.75)	2.15 (0.95)	2.21 (0.88)	37.47	0.002
Very good	930	88.89 (1.18)	5.69 (0.87)	1.34 (0.38)	2.13 (0.44)	1.96 (0.43)		
Good	2576	86.73 (1.41)	7.36 (1.37)	1.76 (0.36)	2.67 (0.33)	1.48 (0.28)		
Fair	3212	89.83 (0.76)	4.8 (0.52)	1.98 (0.29)	2.33 (0.34)	1.06 (0.22)		
Poor	1609	90.65 (0.93)	3.14 (0.52)	2.8 (0.46)	1.83 (0.35)	1.58 (0.36)		
**Region**								
Central	1311	89.65 (1.00)	4.35 (0.75)	2.62 (0.51)	2.45 (0.59)	0.94 (0.33)	64.53	<0.001
East	2593	86.27 (1.35)	6.18 (1.14)	2.7 (0.44)	2.68 (0.37)	2.16 (0.34)		
North-central	1190	91.83 (1.27)	3.96 (0.68)	1.08 (0.47)	1.77 (0.53)	1.36 (0.43)		
North-east	653	90.48 (1.55)	4.89 (1.48)	1.09 (0.60)	2.55 (0.65)	0.99 (0.37)		
North-west	608	92.09 (1.69)	5.79 (1.39)	0.99 (0.41)	0.71 (0.33)	0.42 (0.24)		
South	780	91.18 (1.69)	6.94 (1.61)	0.98 (0.58)	0.78 (0.29)	0.12 (0.12)		
South-west	1423	86.66 (1.39)	4.57 (0.78)	2.72 (0.53)	3.82 (0.71)	2.23 (0.52)		
**Depression**								
No	4303	88.19 (0.89)	6.11 (0.82)	1.93 (0.28)	2.35 (0.28)	1.42 (0.21)	7.84	0.098
Yes	3261	88.98 (0.73)	4.46 (0.47)	2.42 (0.32)	2.61 (0.32)	1.52 (0.22)		
**Working status**								
NO	3447	91.85 (0.59)	3.45 (0.40)	2.29 (0.33)	1.55 (0.27)	0.86 (0.18)	52.90	<0.001
Yes	5111	86.45 (0.87)	6.95 (0.78)	1.79 (0.21)	2.94 (0.29)	1.88 (0.24)		
**Social activities**								
No	4097	89.51 (0.98)	4.68 (0.89)	2.19 (0.31)	2.20 (0.26)	1.42 (0.20)	3.88	0.423
Yes	4461	88.33 (0.69)	5.98 (0.49)	1.86 (0.25)	2.41 (0.26)	1.42 (0.20)		
**Sleep time**								
<6.0 h	2336	89.63 (0.84)	4.10 (0.56)	2.51 (0.39)	2.39 (0.36)	1.37 (0.26)	9.75	0.283
6.0~7.9 h	2847	87.81 (0.89)	6.26 (0.79)	2.04 (0.33)	2.44 (0.34)	1.46 (0.24)		
≥8.0 h	2894	88.46 (1.14)	5.49 (1.05)	1.72 (0.29)	2.61 (0.33)	1.72 (0.28)		

Drs/wk: drinks/week

There were also more occasional drinkers among older women. Women who were married, or had high levels of education or good health status also tended to be former drinkers. The biggest proportion of heavy drinkers was in those living in the south-west. Those without a work trended to be lifetime abstainers.

### Factors associated with alcohol consumption

The results of logistic regression detecting the relationship between included factors and alcohol consumption were listed in Tables 4 and 5. Among men, age and health situation were significantly and inversely associated with all alcohol consumption behaviors including current drinking, binge drinking, heavy drink and daily alcohol consumption. Smoking and living in north-east had a positive association with all of the alcohol consumption behaviors. Current working increased the risk of alcohol consumption behaviors including current drinking, heavy drink and daily alcohol consumption and social activities associated with current drinking (Table 4). Among women, smoking and current working were associated with increased levels of alcohol consumption. Compared with those in the central region, those living in the east area had an increased risk of drinking (Table 5).

**Table 4:** The relationships between potential factors and different alcohol consumption behaviors using logistic regression models with corresponding odds ratio and 95% confidence intervals in men

	Alcohol consumption behaviors			
	**Current drinking**	**Binge drinking**	**Heavy drinking**	**Daily alcohol consumption**
**Age (years)**				
45~49	1.00	1.00	1.00	1.00
50~54	1.05 (0.86, 1.28)	1.01 (0.76, 1.35)	1.08 (0.84, 1.40)	1.17 (0.88, 1.55)
55~59	1.01 (0.80, 1.27)	0.96 (0.60, 1.53)	1.03 (0.83, 1.28)	1.23 (0.90, 1.69)
60~64	0.87 (0.71, 1.06)	0.79 (0.59, 1.06)	1.12 (0.88, 1.43)	1.48 (1.14, 1.91) ^*^
65~69	0.74 (0.59, 0.94)^*^	0.48 (0.34, 0.67) ^*^	0.91 (0.69, 1.22)	1.30 (0.95, 1.76)
70~74	0.49 (0.36, 0.67) ^*^	0.27 (0.18, 0.43) ^*^	0.58 (0.41, 0.83) ^*^	0.93 (0.64, 1.37)
≥75	0.63 (0.46, 0.86) ^*^	0.20 (0.12, 0.35) ^*^	0.59 (0.41, 0.85) ^*^	0.93 (0.65, 1.34)
**Rural-urban status**				
Rural	1.00	1.00	1.00	1.00
Urban	1.05 (0.86, 1.30)	1.15 (0.92, 1.43)	0.82 (0.64, 1.07)	0.92 (0.71, 1.20)
**Marital status**				
Separated/divorced/widowed/ never been married	1.00	1.00	1.00	1.00
Married	0.92 (0.51, 1.65)	0.60 (0.32, 1.12)	0.57 (0.29, 1.12)	1.07 (0.54, 2.11)
**Living alone**				
No	1.00	1.00	1.00	1.00
Yes	1.06 (0.67, 1.69)	0.76 (0.43, 1.35)	0.67 (0.36, 1.23)	1.23 (0.70, 2.16)
**Education**				
Illiteracy	1.00	1.00	1.00	1.00
Primary or junior high school	1.04 (0.83, 1.31)	0.83 (0.63, 1.10)	0.91 (0.70, 1.18)	1.08 (0.84, 1.39)
Senior high school	1.06 (0.83, 1.35)	0.90 (0.67, 1.21)	0.87 (0.65, 1.16)	1.07 (0.81, 1.39)
Graduate	1.18 (0.88, 1.58)	1.27 (0.90, 1.80)	1.00 (0.72, 1.39)	0.89 (0.66, 1.21)
**Smoking**				
No	1.00	1.00	1.00	1.00
Yes	2.18 (1.82, 2.61) ^*^	2.27 (1.82, 2.83) ^*^	2.44 (1.99, 3.00) ^*^	2.30 (1.89, 2.81) ^*^
**Self- Comment of Your Health**				
Excellent	1.00	1.00	1.00	1.00
Very good	0.81 (0.55, 1.17)	1.16 (0.57, 2.37)	0.98 (0.61, 1.55)	0.55 (0.28, 1.06)
Good	0.68 (0.47, 1.00)	0.95 (0.51, 1.76)	0.72 (0.39, 1.34)	0.50 (0.27, 0.94) ^*^
Fair	0.51 (0.33, 0.79) ^*^	0.79 (0.47, 1.35)	0.60 (0.33, 1.12)	0.40 (0.21, 0.75) ^*^
Poor	0.40 (0.26, 0.61) ^*^	0.45 (0.24, 0.82) ^*^	0.46 (0.25, 0.84) ^*^	0.30 (0.16, 0.57) ^*^
**Region**				
Central	1.00	1.00	1.00	1.00
East	1.41 (1.10, 1.80) ^*^	1.22 (0.91, 1.65)	1.58 (1.18, 2.12) ^*^	1.77 (1.31, 2.40) ^*^
North-central	0.92 (0.69, 1.22)	1.31 (0.94, 1.84)	0.94 (0.66, 1.35)	0.99 (0.69, 1.41)
North-east	1.89 (1.43, 2.51) ^*^	1.59 (1.09, 2.31) ^*^	1.86 (1.29, 2.68) ^*^	1.84 (1.26, 2.68) ^*^
North-west	0.48 (0.34, 0.67) ^*^	0.81 (0.52, 1.26)	0.21 (0.12, 0.35)	0.16 (0.09, 0.27) ^*^
South	0.46 (0.26, 0.81)	0.66 (0.33, 1.30)	0.74 (0.37, 1.49)	0.47 (0.28, 0.81) ^*^
South-west	1.13 (0.85, 1.48)	0.98 (0.69, 1.39)	1.48 (1.07, 2.04) ^*^	1.45 (1.05, 2.01) ^*^
**Depression**				
No	1.00	1.00	1.00	1.00
Yes	0.94 (0.81, 1.09)	1.01 (0.83, 1.22)	0.90 (0.76, 1.06)	0.86 (0.72, 1.01)
**Working status**				
NO	1.00	1.00	1.00	1.00
Yes	1.49 (1.26, 1.75) ^*^	1.30 (0.92, 1.85)	1.35 (1.11, 1.64) ^*^	1.37 (1.06, 1.78) ^*^
**Social activities**				
No	1.00	1.00	1.00	1.00
Yes	1.14 (1.01, 1.30) ^*^	1.16 (0.98, 1.38)	1.04 (0.89, 1.22)	0.96 (0.83, 1.12)
**Sleep time**				
<6.0 h	1.00 (0.82, 1.22)	0.96 (0.74, 1.24)	1.06 (0.83, 1.34)	1.06 (0.87, 1.29)
6.0~7.9 h	1.00	1.00	1.00	1.00
≥8.0 h	0.86 (0.73, 1.00)	0.90 (0.74, 1.09)	1.05 (0.86, 1.27)	1.02 (0.87, 1.21)

Model were adjusted for age groups, rural-urban status, marital status, living status, education, smoking status, self- comment of health, region, depression, working status, social activities and sleep time

^*^, *P*<0.05

**Table 5:** The relationships between potential factors and different alcohol consumption behaviors using logistic regression models with corresponding odds ratio and 95% confidence intervals in women

	Alcohol consumption behaviors			
	**Current drinking**	**Binge drinking**	**Heavy drinking**	**Daily alcohol consumption**
**Age (years)** ^**a**^				
45~49	1.00	1.00	1.00	1.00
50~54	1.41 (0.89, 2.23)	0.23 (0.04, 1.25)	1.78 (0.70, 4.49)	1.73 (0.64, 4.71)
55~59	1.50 (1.00, 2.25)	1.06 (0.38, 2.96)	2.38 (1.26, 4.48) ^*^	2.44 (1.25, 4.77) ^*^
60~64	1.83 (1.18, 2.83) ^*^	2.07 (0.69, 6.17)	3.34 (1.71, 6.54) ^*^	3.42 (1.72, 6.81) ^*^
65~69	1.61 (0.95, 2.71)	0.38 (0.10, 1.44)	1.97 (0.89, 4.35)	1.88 (0.83, 4.26)
70~74	1.31 (0.70, 2.46)	-	1.28 (0.45, 3.65)	1.49 (0.54, 4.10)
≥75	1.75 (0.90, 3.40)	-	3.54 (1.43, 8.75) ^*^	3.77 (1.63, 8.73) ^*^
**Rural-urban status**				
Rural	1.00	1.00	1.00	1.00
Urban	0.86 (0.56, 1.34)	4.23 (1.57, 11.38) ^*^	0.63 (0.31, 1.28)	0.49 (0.21, 1.12)
**Marital status**				
Separated/divorced/widowed/ never been married	1.00	1.00	1.00	1.00
Married	0.93 (0.52, 1.66)	0.83 (0.21, 3.20)	0.62 (0.23, 1.66)	0.75 (0.29, 1.96)
**Living alone**				
No	1.00	1.00	1.00	1.00
Yes	1.20 (0.74, 1.94)	1.85 (0.48, 7.09)	0.92 (0.36, 2.30)	1.06 (0.44, 2.55)
**Education**				
Illiteracy	1.00	1.00	1.00	1.00
Primary or junior high school	1.12 (0.83, 1.51)	0.24 (0.08, 0.71) ^*^	1.14 (0.69, 1.89)	1.30 (0.74, 2.27)
Senior high school	0.89 (0.55, 1.45)	0.54 (0.15, 1.97)	0.93 (0.42, 2.08)	0.89 (0.36, 2.17)
Graduate	1.71 (0.99, 2.96)	1.06 (0.35, 3.26)	2.19 (0.85, 5.67)	2.07 (0.68, 6.32)
**Smoking**				
No	1.00	1.00	1.00	1.00
Yes	3.85 (2.69, 5.52) ^*^	7.17 (3.08, 16.69) ^*^	6.33 (3.95, 10.15) ^*^	5.99 (3.63, 9.88) ^*^
**Self- Comment of Your Health**				
Excellent	1.00	1.00	1.00	1.00
Very good	0.98 (0.50, 1.95)	1.02 (0.22, 4.63)	0.84 (0.32, 2.22)	0.81 (0.28, 2.29)
Good	0.98 (0.48, 1.98)	0.96 (0.19, 4.78)	0.66 (0.25, 1.76)	0.70 (0.25, 1.94)
Fair	0.77 (0.38, 1.57)	0.55 (0.10, 3.03)	0.47 (0.16, 1.36)	0.46 (0.15, 1.40)
Poor	0.72 (0.33, 1.54)	0.25 (0.04, 1.56)	0.66 (0.22, 1.97)	0.64 (0.20, 2.07)
**Region** ^**a**^				
Central	1.00	1.00	1.00	1.00
East	1.63 (1.03, 2.58) ^*^	0.80 (0.23, 2.79)	2.06 (0.97, 4.38)	1.90 (0.89, 4.03)
North	0.75 (0.45, 1.25)	0.79 (0.23, 2.80)	0.72 (0.31, 1.70)	0.56 (0.23, 1.34)
South	1.04 (0.62, 1.74)	0.68 (0.13, 3.48)	1.07 (0.47, 2.46)	1.23 (0.53, 2.86)
**Depression**				
No	1.00	1.00	1.00	1.00
Yes	1.24 (0.91, 1.68)	1.81 (0.73, 4.52)	1.06 (0.64, 1.76)	1.06 (0.60, 1.85)
**Working status**				
NO	1.00	1.00	1.00	1.00
Yes	2.36 (1.70, 3.26) ^*^	4.55 (2.08, 9.94) ^*^	2.87 (1.76, 4.66) ^*^	2.15 (1.30, 3.58) ^*^
**Social activities**				
No	1.00	1.00	1.00	1.00
Yes	1.26 (0.99, 1.60)	1.31 (0.60, 2.89)	1.11 (0.75, 1.64)	1.04 (0.71, 1.53)
**Sleep time**				
<6.0 h	0.91 (0.64, 1.29)	0.42 (0.14, 1.27)	0.82 (0.49, 1.38)	0.81 (0.50, 1.33)
6.0~7.9 h	1.00	1.00	1.00	1.00
≥8.0 h	1.08 (0.79, 1.47)	1.02 (0.40, 2.58)	1.02 (0.64, 1.65)	1.06 (0.65, 1.73)

Model were adjusted for age groups, rural-urban status, marital status, living status, education, smoking status, self- comment of health, region, depression, working status, social activities and sleep time

^*^, *P*<0.05

^a^ Several categories in the statistical analysis were merged because of a small number of observations

### The type of alcohol beverages

The favorite type of alcohol was spirits among both men and women. As age increased, the proportion of spirits consumed increased, and the proportion of beer consumed decreased. There were no significant differences in distribution of alcoholic type between urban or rural areas. As education level increased, participants were less likely to drink spirits. The proportion of spirits drunk was lowest in people living in the eastern area, where the consumption of both beer and wine was higher. Those with current working were more likely to drink beer and ≥ 2 types of alcohol beverage (Table 6).

**Table 6:** Distribution (percentage) of preferred alcoholic beverages consumed by current drinkers

	Men						Women					
	Sprit	Beer	Wine	≥ 2 types	χ^2^	*P* value	Sprit	Beer	Wine	≥ 2 types	χ^2^	*P* value
**Age (years)**												
45~49	44.67	20.96	0.52	33.85	98.86	<0.001^*^	29.17	44.44	0	26.39	8.70	0.034^*^
50~54	53.56	18.2	0	28.24			32.2	38.98	1.69	27.12		
55~59	57.92	13.92	0.16	28			54.65	22.09	3.49	19.77		
60~64	60.56	13.08	0.2	26.16			54.93	22.54	2.82	19.72		
65~69	68.33	10	0.33	21.33			63.16	21.05	0	15.79		
70~74	66.26	11.66	1.23	20.86			60.71	28.57	0	10.71		
≥75	71.86	8.98	1.8	17.37			76.67	13.33	3.33	6.67		
**Rural-urban status**												
Rural	57.27	15.54	0.23	26.97	6.051	0.109	49.69	29.81	0.93	19.57	11.91	0.008^*^
Urban	57.81	13.62	0.83	27.74			46.43	21.43	7.14	25		
**Marital status**												
Separated/divorced/widowed/ never been married	68.83	10.39	0	20.78	14.33	0.003^*^	51.61	22.58	4.84	20.97	4.74	0.192
Married	56.28	15.54	0.43	27.75			49.07	29.81	1.24	19.88		
**Living alone**												
No	56.42	15.57	0.4	27.61	7.40	0.060	48.81	29.49	1.69	20	0.55	0.908
Yes	64.11	11.66	0.31	23.93			51.69	25.84	2.25	20.22		
**Education**												
Illiteracy	66.03	11.75	0	22.22	28.43	<0.001^*^	56.89	23.35	2.4	17.37	14.8581	0.095
Primary or junior high school	59.63	14.34	0.32	25.71			45.99	33.58	0.73	19.71		
Senior high school	54.45	16.54	0.38	28.63			42.86	35.71	0	21.43		
Graduate	50	17.09	0.85	32.05			36.84	26.32	5.26	31.58		
**Smoking**												
No	48.55	23.21	0.39	27.85	36.10	<0.001^*^	47.25	31.39	1.94	19.42	6.19	0.103
Yes	59.3	13.29	0.39	27.02			58.67	17.33	1.33	22.67		
**Self- Comment of Your Health**												
Excellent	54.81	17.04	0	28.15	15.67	0.207	40	40	0	20	16.24	0.180
Very good	53.22	16.41	0.44	29.93			37.74	39.62	0	22.64		
Good	56.31	15.99	0.4	27.31			58.4	22.4	3.2	16		
Fair	58.61	13.29	0.33	27.78			48.44	28.13	2.34	21.09		
Poor	64.12	14.95	0.66	20.27			46.03	30.16	0	23.81		
**Region**												
Central	58	11.5	0	30.5	220.76	<0.001^*^	48.94	25.53	0	25.53	77.88	<0.001^*^
East	46.34	21.64	0.79	31.23			36.18	38.16	1.97	23.68		
North-central	63.71	12.27	0	24.02			44.74	36.84	0	18.42		
North-east	51.62	20.22	0	28.16			28	52	4	16		
North-west	47.37	9.65	0	42.98			54.55	0	0	45.45		
South	53.21	12.84	2.75	31.19			58.33	8.33	25	8.33		
South-west	79.69	6.19	0	14.12			75.76	12.12	0	12.12		
**Depression**												
No	55.78	15.89	0.36	27.97	6.97	0.073	46.07	30.37	3.14	20.42	3.98	0.264
Yes	61.13	13.81	0.13	24.93			51.57	27.04	0.63	20.75		
**Working status**												
NO	66.73	12.04	1.42	19.82	48.36	<0.001^*^	59.79	24.74	3.09	12.37	8.45	0.038^*^
Yes	54.96	15.89	0.13	29.02			45.99	29.97	1.39	22.65		
**Social activities**												
No	61.73	13.82	0.15	24.3	22.28	<0.001^*^	53.01	26.78	1.64	18.58	1.75	0.626
Yes	53.44	16.25	0.6	29.7			46.27	30.35	1.99	21.39		
**Sleep time**												
<6.0 h	60.35	14.39	0.35	24.91	9.60	0.142	54.29	25.71	2.86	17.14	3.76	0.709
6.0~7.9 h	55.03	16.7	0.66	27.61			47.66	27.34	1.56	23.44		
≥8.0 h	57.93	14.08	0.17	27.82			47.33	32	1.33	19.33		

^*^, *P*<0.05

## Discussion

Approximately 36.42% of men and 3.73% of women reported consuming alcohol in the previous 12 months. The findings on drinking prevalence in our study are consistent with previous baseline survey from the China Kadoorie Biobank which is a nationwide prospective blood-based cohort study [15, 16]. The study involving 512,000 participants aged 30-79 years found a weekly drinking prevalence of 33% in men and 2% in women in 2004-08 [15, 16]. The estimated prevalence in our study was also comparable to the results from CLHLS among 16,255 adults aged ≥65 years. The study found that 34.14% of men reported drinking alcohol most weeks, compared with only 7.20% of women [17]. The prevalence among men was lower than that reported in the 2010–2012 Chinese Nutrition and Health Survey. The probable reason for this difference was that the age ranges of study participants varied. In Li’s study, the participants were aged 18 years or over, and the drinking rate was much higher among young adults than older people [5]. Generally, the alcohol consumption levels in China were lower than Whites or developed countries [17, 18]. The differences between the prevalence of alcohol consumption in China and developed countries may be partially due to the different levels of economic development. It was reported that greater economic wealth was broadly associated with higher levels of alcohol consumption and lower abstention rates [9]. Despite the relative low level of alcohol consumption, planning and implementation of a public health approach to alcohol control is urgently recommended for China’s governments because of a steady increase in alcohol production and consumption in recent decades [4] and the burden attributed to alcohol consumption [19–22].

Gender differences in drinking behavior have been observed in many studies [23, 24]. Drinking and smoking have been traditionally accepted and expected behaviors for Chinese men, but not for Chinese women. Research showed that 31.4% of female non-drinkers listed restriction by society as the main reason for their abstinence, whereas “Feeling sick after drinking” are the most common reason for male non-drinkers [25]. In China, especially in rural areas, men are more likely to take part in social activities, and it is accepted for men to drink with a meal, or to reduce pressure from work or to liven up the atmosphere. The highest prevalence of current drinking occurred during middle age in men. This is the age when men tend to be at the peak of their careers and might have more social interaction, which could increase the probability of drinking alcohol. It may also explain why the current drinking rate was higher in women with current work. Women with work tend to have more income and higher social status and therefore more social interactions.

Tobacco users were more likely to have other unhealthy dietary and health behaviors. Male drinkers who smoked also drank more alcohol than those who did not. Alcohol and tobacco are social tools in China. Traditionally, men believe that giving a cigarette and drinking together can bring people closer together quickly. Alcohol and tobacco use are highly co-morbid and have interactive effects on alcohol- and tobacco-motivated behaviors [26, 27]. Alcohol potentiates smoking behavior and vice versa.

Several variables such as depression, poor health status and sleeping problems, commonly co-occur with alcohol use disorder, and alcohol use disorder might increase the risk of those health-related disorders [28, 29]. However, we did not found the link between depression, sleep time and alcohol behaviors. The assessment of depression was concluded with the 10-item CES-D rather than diagnosed by psychologists, which may lead to inaccuracies. Furthermore, the purpose of social drinking in China is mainly to establish a joyful atmosphere and to easing tension [30]. Meanwhile, most drinking takes place accompanied by a meal which alleviated the damage done to the central nervous system by alcohol. Participants with poor health seemed to drink less. This may be because of the “sick-quitter” effect, where those with poor health stop drinking [31].

The proportion of heavy drinking in rural areas was higher than that in urban areas, although the difference was not statistically significant. Previous studies in China validated that participants who lived in rural areas drank more alcohol including all types (liqueur, beer and wine) than participants who lived in urban areas [32]. In Liu et al’s study, rural residents had higher risk of daily alcohol drinking and reporting alcohol dependence, regardless of province [3]. Meanwhile, homemade alcohol that was unrecorded alcohol was common in rural China. These findings claim the problems of alcohol consumption in rural areas and it deserves more attention from the Chinese government to take active measures for alcohol consumption control.

Alcohol consumption behaviors and lifestyles vary greatly across different regions in China. In this study, men in north-east region consumed more alcohol than residents of other areas. This may be because of the cold weather and the drinking culture in the north-east areas. However, women living in east region were more likely to drink. Probable reason is that east region is economically developed region of China, the social status and employment rate of women is comparable to that of men and they therefore have more access to social drinking.

The most common type of alcoholic beverage drunk was spirits. Individuals of different ages tended to consume different types of alcohol. Both men and women were more likely to consume spirits when they were older. The second most popular beverage was beer. Beer consumption trends with age showed the opposite of those for spirits. We also found a relatively high number of people who drank several types of beverage, particularly among younger adults. This may be because beer is relatively inexpensive and popular among young adults [3]. This phenomenon of mixed drinking is probably the result of exposure to a wider availability of alcoholic beverages brought by the rapid economic development and westernization of China [15].

Alcohol-related data have been reported in several studies of CHARLS [33–35]. However the focus of these researches were not with a view to alcohol consumption and the alcohol-related analyses were not exhaustive. The advantages of this study are the data adopted are based on national survey data, which is representative for the Chinese population and multi-dimensional data of alcohol consumption including alcohol type, drinking frequency and volume, and drinking pattern were analyzed. However, several limitations should be considered. Firstly, the data was from baseline survey that was cross-sectional designed, and therefore we only described the potential factors related to drinking behaviors and we cannot make any causal inferences about those factors for drinking behaviors. Secondly, we used a self-administered questionnaire to collect information on alcohol consumption and this may have resulted in self-report bias. Thirdly, the measure of alcohol use based on “the last time you consumed alcohol” may not accurately collect individual data on alcohol consumption [36, 37]. Lastly, the information was collected in CHARLS 2011–2012, and therefore the prevalence may now be different. Alcohol consumption has rapidly increased over time, and therefore further research is needed to monitor drinking patterns across the country.

## Conclusion

There were strong gender disparities in drinking rate. Alcohol consumption behaviors and alcoholic type was significantly influenced by socio- cultural-demographic factors, such as age, smoking, working status and different regions. Our findings suggest that Chinese government should be provided with multidimensional interventions to meet different demands for different populations. In the future, the socio-cultural-demographic factors affecting alcohol patterns should be further focused on to promote the development of alcohol control strategies.

## Data Availability

The datasets generated and analyzed during the current study are available in the CHARLS website, available in http://charls.pku.edu.cn/en.
